# *In vivo* knee joint friction after total knee arthroplasty is highly dynamic and phase-dependent

**DOI:** 10.3389/fbioe.2026.1885711

**Published:** 2026-08-04

**Authors:** Julian N. Zierke, Philippe Moewis, Rainald M. Ehrig, William R. Taylor, Adam Trepczynski, Georg N. Duda, Philipp Damm

**Affiliations:** 1 Julius Wolff Institute, Berlin Institute of Health at Charité – Universitätsmedizin Berlin, Berlin, Germany; 2 Zuse Institute Berlin (ZIB), Berlin, Germany; 3 Institute for Biomechanics, ETH Zürich, Zürich, Switzerland

**Keywords:** gait assessment, *in vivo* friction, knee joint friction, knee joint kinematics, single plane fluoroscopy, total knee arthroplasty

## Abstract

**Introduction:**

Natural synovial knee joints exhibit very low friction, enabling efficient long-term function. In contrast, total knee arthroplasty (TKA) introduces artificial articulating surfaces with altered kinematic and tribological conditions. However, how *in vivo* friction evolves across different dynamic activities after TKA remains unclear. The aim of this study was to quantify *in vivo* knee joint friction after TKA and to investigate its relationship with activity-dependent loading conditions and knee joint kinematics.

**Methods:**

*In vivo* joint friction was assessed in three subjects with instrumented telemetric knee implants during chair sit, squat, and level walking. Joint contact forces and moments were recorded *in vivo*, while synchronous fluoroscopic imaging was used to determine knee joint kinematics. Loading data were referenced to the instantaneous axis of rotation to calculate frame-by-frame knee joint friction throughout each activity.

**Results:**

*In vivo* friction after TKA was highly dynamic and activity-dependent, showing distinct temporal variations across the three different activities. During chair sit and squat, coefficients of friction (µ) of up to approximately 0.15 were observed during loaded flexion and extension phases, consistent with mixed or boundary lubrication regimes reported *in vitro*. In contrast, substantially higher µ values of up to 0.3 during chair sit and 0.43 during gait were observed during low-loading or rapid kinematic transition phases. Peak friction moments reached up to 5.5 Nm during chair sit, 5.0 Nm during squat, and 8.9 Nm during gait. Overall, friction behavior varied substantially throughout the movement cycle and exhibited pronounced phase-dependent and inter-individual variability with peaks during swing phases.

**Discussion:**

These findings demonstrate that *in vivo* knee joint friction after TKA is dynamic, activity- and phase-dependent. The observed friction dynamics illustrate that friction is only partially linked to contact load magnitude, while motion velocity and possible lubrication-related effects may also influence friction. These findings may help improve experimental and computational models used in TKA tribology and implant evaluation.

## 1 Introduction

Natural human synovial joints exhibit highly efficient tribological behavior characterized by low friction, thereby minimizing wear and supporting long-term joint function. This remarkable performance results from the complex interaction between articulating tissues, particularly cartilage, and synovial fluid, which together maintain effective joint lubrication. Although *in vivo* friction behavior has not yet been directly quantified, these interactions are assumed to operate under low-friction conditions based on measurements obtained *in vitro* (Unsworth et al., 1975; Merkher et al., 2006; Forster and Fisher, 1996).

After total knee arthroplasty (TKA), the friction behavior will be different from the one in a natural articulation due to the exchange of natural surfaces by an interaction of metal, ceramic or plastic materials with fundamentally different tribological properties. However, these *in vivo* friction properties for such artificial articulations have not been sufficiently characterized due to a lack of existing data. The current knowledge of knee joint friction properties has been derived primarily from *in vitro* studies, which often involve unrealistic loading and kinematic conditions, as well as simplified implant contact scenarios (Galetz et al., 2010; Mazzucco and Spector, 2004; Scholes and Unsworth, 2006). Furthermore, *in silico* models and 3D implant design processes often represent friction as a constant parameter during motion, typically by assuming a constant Coulomb friction coefficient (Godest et al., 2002; Halloran et al., 2005; K et al., 2007; Mell et al., 2018; O’Brien et al., 2013). As a result, realistic *in vivo* friction variations and their relationship to knee joint kinematics remain insufficiently characterized.

While total knee arthroplasty (TKA) exhibits complex kinematic behavior, it is reasonable to assume that the interplay between the kinematics and kinetics may play a dominant role in frictional behavior during daily activities. A better assessment of these friction dynamics could provide valuable insights into activity-driven changes in TKA and eventually guide usage of TKA but also its design and pre-clinical testing in a more accurate manner.

Between the articulating surfaces, synovial fluid acts as lubricant and eventually as shock absorber, exhibiting strong non-linear and velocity-dependent shear-thinning properties as a non-Newtonian fluid (Cooke et al., 1978; Schurz and Ribitsch, 1987; Herbster et al., 2021). It is assumed that this lubrication mechanisms are altered in joints affected by osteoarthritis or rheumatoid arthritis (Cooke et al., 1978; Schurz and Ribitsch, 1987; Herbster et al., 2021; Balazs et al., 1967) and total knee arthroplasty (TKA) (Herbster et al., 2021; Mazzucco et al., 2002) in an unknown manner.

The aim of this study was to quantify *in vivo* friction after total knee arthroplasty and to investigate its relationship with activity-dependent loading conditions and knee joint kinematics. We hypothesize that knee joint friction after total knee arthroplasty is not constant but varies with activity-dependent loading and joint kinematics.

## 2 Methods

### 2.1 Instrumented knee implant

*In vivo* forces and moments were measured using an instrumented knee implant (Heinlein et al., 2007). The knee implant is a posterior cruciate sacrificing total knee arthroplasty (TKA) (Zimmer, Switzerland; INNEX type FIXUC) with a symmetrical ultra-congruent polyethylene (UHMWPE) inlay, size M10. The femoral component is made of cobalt-chrome (CoCr) and is sized M+ for males and M for females. The tibial component, also made of CoCr, is implanted with an individual posterior slope (7°–11°). The telemetry system allows the measurement of three contact forces and joint moments (as a sum of friction and bending) relative to the deepest midpoint in the inlay. The forces and moments are presented in the tibial coordinate system (Kutzner et al., 2010; Taylor et al., 2017) with a mean measurement error below 2% (Heinlein et al., 2007). The positive components $F_{x}$ , $F_{y}$ and $F_{z}$ act in lateral, anterior, and superior directions, respectively. Force components $F_{y}$ and $F_{z}$ act together in the sagittal plane and were used to determine the resultant sagittal joint contact force ${\vec{F}}_{YZ}$ . The measured moments $M_{x}$ , $M_{y}$ , and $M_{z}$ act clockwise around their positive axes. All forces are reported in N and moments in Nm. Data points for forces and moments were only used if they had a corresponding fluoroscopic frame. Detailed descriptions of the telemetry (Heinlein et al., 2007; Bergmann et al., 2008; Graichen et al., 2007) and the external measurement systems have been published previously (Taylor et al., 2017). This study was approved by the Ethics Committee of Charité–Universitätsmedizin Berlin, Germany (EA4/069/06) and ETH Zürich, Switzerland (EK 2013-N-90).

### 2.2 Subjects and measurement

**FIGURE 1:**
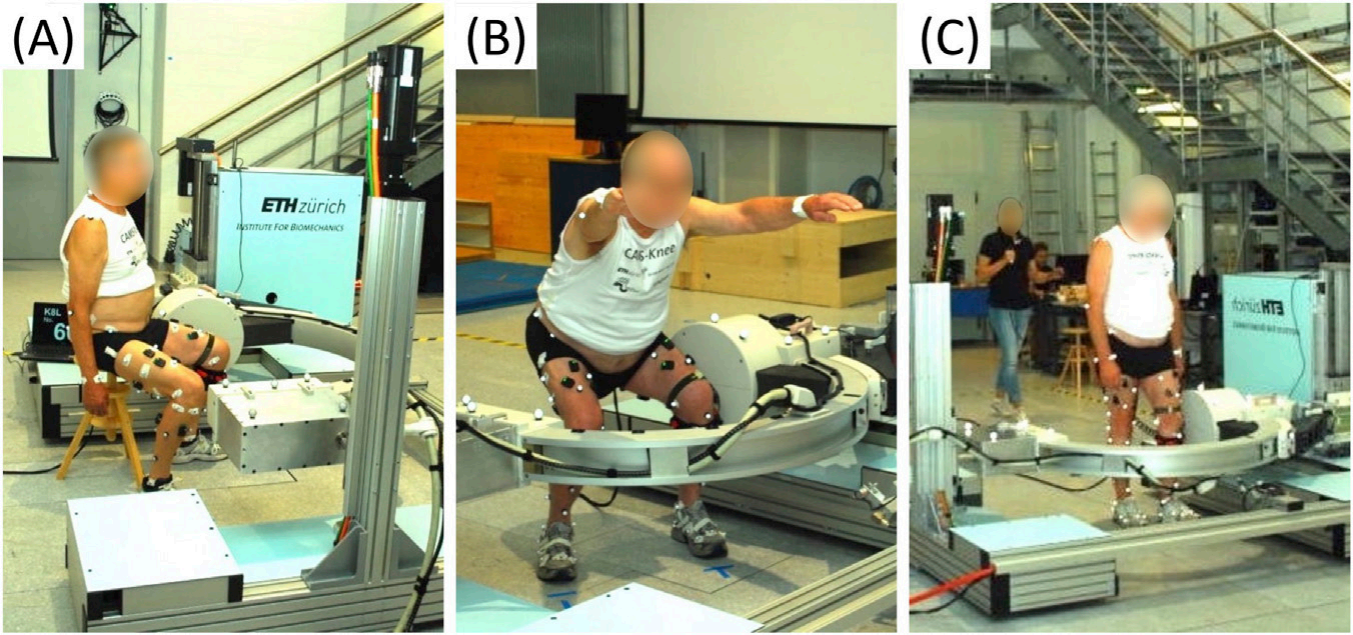
Performed activities: **(A)** chair sit, **(B)** squat and **(C)** gait, including the experimental setup for synchronized measurements using fluoroscopy and instrumented knee implants.

In this study, the CAMS Knee dataset was analyzed (cams-knee.orthoload.com). However, only participants with complete, high-quality, and synchronized load and fluoroscopic kinematic data for all investigated activities were included in the present analysis - three out of six (Table 1). The measurements took place 64–69 months postoperatively. *In vivo* knee joint loads were measured while synchronized fluoroscopic images were acquired (Figure 1) using a single plane C-arm fluoroscopy system (Philips Medical Systems, Switzerland) with a frame rate of 25 Hz (Taylor et al., 2017).

**TABLE 1:** Participant anthropometric data.

Participant	K5R	K7L	K8L
Sex	m	f	m
Height (cm)	174	165	175
Bodyweight (N)	938	653	773
BMI (kg/m^2^)	32	24	26
Age (y)	65	80	76
Months post-op	69	67	64
Implant inlay size	M10	M10	M10
Tibial component	Instrumented knee implant		
Femoral component	M+	M	M+
Posterior tibial slope (°)	7	7	11

The activities performed were chair sit, squat, and gait (level walking). All tasks involved controlled movement patterns, which facilitate reproducible measurements of loads and motions and allow a consistent analysis of the resulting friction data. Based on our hypothesis that knee joint friction after total knee arthroplasty is not constant but varies with activity-dependent loading and knee joint kinematics, we included activities with distinct loading and kinematic profiles. Two high-flexion activities were assessed: stand-up/sit-down from a chair and a squat. The stand-up/sit-down task involved reaching maximal knee extension before returning to the seated position, with chair height standardized across participants. Chair sit movements include high quadriceps activity but also phases of minimal joint loading (Kutzner et al., 2010), whereas squat involves similar flexion–extension motion but different loading characteristics due to sustained high joint forces. The squat activity started from full knee extension with hands extended forward, followed by maximal active knee flexion and a return to extension. Gait was included as a representative highly dynamic daily activity, differing not only in loading but also in flexion–extension kinematics, and was performed at each participant’s self-selected speed.

### 2.3 *In vivo* implant kinematic and load transformation

Three dimensional (3D) models of the implant components were registered to the fluoroscopic images (Taylor et al., 2017; Trepczynski et al., 2021). Registration errors, assessed for a similar TKA, were 0.15°/0.3 mm for in-plane and 0.25°/1 mm for out-of-plane (List et al., 2017). This technique provides a mathematical representation of the 3D position and orientation of the tibial and femoral components relative to the laboratory or fluoroscopy coordinate system (Taylor et al., 2017; Grood and Suntay, 1983). After registering the 3D models, smoothed 3D transformations were computed. Based on these transformations, the positions and orientations of the femoral relative to the tibial component were calculated, enabling determination of the flexion angles in the sagittal plane (α), with the posterior tibial slope accounted for and removed. The absolute angular velocity (ω) was subsequently calculated as the time derivative of the flexion–extension angle. The absolute tibiofemoral sliding velocity at the approximated contact point was subsequently estimated by multiplying the absolute angular velocity by the magnitude of the effective friction lever arm $\left|{\vec{r}}_{fric}\right|$ ( $v_{slide}=\omega \cdot \left|{\vec{r}}_{fric}\right|$ ).

**FIGURE 2:**
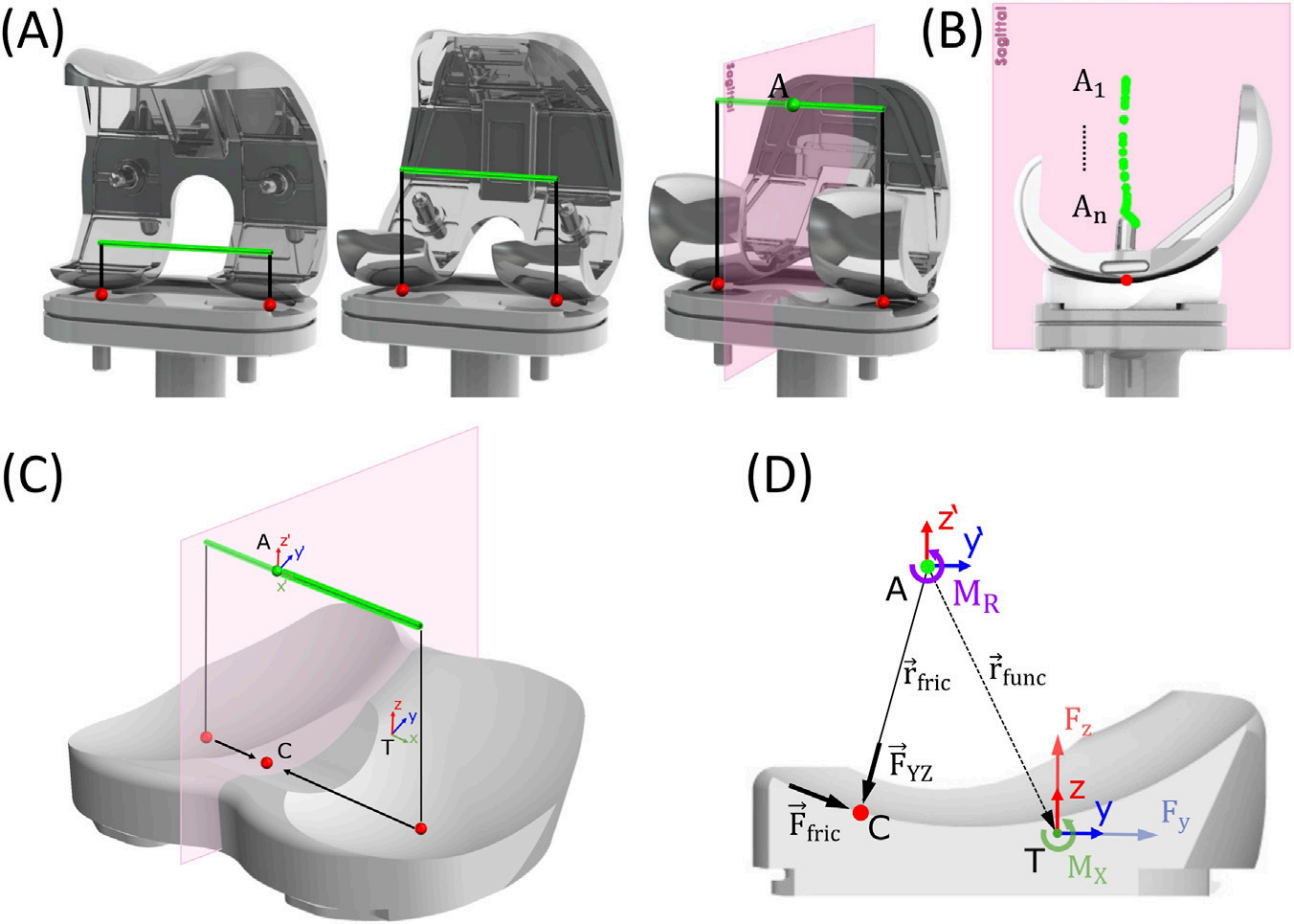
**(A)** Determination and variation in position of the functional axis (green) using the femoral curvature center at deepest femoral points (red spheres) from flexion to extension (left to right) during the squat activity; **(B)** Illustrated history of variation in position of the cutting point “A” (green circle) between the axis and the sagittal plane as well as deepest point (red circle) position during flexion; **(C)** Approximation of the contact point C; **(D)** Free body diagram in the sagittal plane through the origin of measurement coordinate system.

The geometrical curvature of the medial and lateral femoral condyles was extracted from the computer aided design (CAD) models of the implant and used to identify the corresponding deepest points relative to the tibial component coordinate system (CS_T_), defined as a stationary reference, at each time frame. Based on the centers of curvature of the medial and lateral condyles, the functional axis of rotation was determined for each fluoroscopic frame to track positional changes throughout the movement. The intersection of this functional axis with the sagittal plane was defined as the instantaneous point of rotation for flexion–extension in the sagittal plane (cutting point A, Figure 2). The position of this rotation point is represented by the vector ${\vec{r}}_{func}$ , pointing from point A to the origin of CS_T_.

To determine the joint friction acting in the sagittal plane, a 3D moment equilibrium was established about the instantaneous point of rotation A. For the moments about point A to be balanced, the sum of the measured joint moment ${\vec{M}}_{T}$ , the force-induced moment ( ${\vec{r}}_{func}\times {\vec{F}}_{T}$ ), and the friction moment ${\vec{M}}_{Friction}$ must equal zero (Equation 1). Under this condition, the friction moment acting in the sagittal plane corresponds to the x-component of the resulting 3D friction moment, as it represents rotation about the flexion–extension axis. The scalar friction moment $M_{Friction\_x}$ or $M_{R}$ is therefore calculated from the transformed load components (Equation 2) and is defined as positive during joint extension and negative during flexion.

$$\sum {\vec{M}\;}^{\left(A\right)}=\vec{0}={\vec{M}}_{Friction}+{\vec{M}}_{T}+{\vec{r}}_{func}\times {\vec{F}}_{T}$$ (1)

$${M_{Friction\_x}=M_{R}=-M}_{x}-r_{y}F_{z}+r_{z}F_{y}$$ (2)

### 2.4 Dynamic characterization of the joint friction *in vivo*

To characterize the changes in joint friction over time, the coefficient of friction ( μ ) were determined. First, we calculated the magnitude of the contact force ${\vec{F}}_{YZ}$ including the components $F_{y}$ and $F_{z}$ . Next, the current contact point (point C) was estimated at each timepoint from the fluoroscopic data. This is achieved by using the sagittal intersection point at x = 0 of the connecting line between the medial and lateral deepest points. This instantaneous point of contact was represented by ${\vec{r}}_{fric}$ . Subsequently, the coefficient of friction in the sagittal plane was calculated using the previously calculated friction moment and the Coulomb friction law, with the sagittal contact force as normal force (Equations 3, 4).

$$\left|M_{R}\right|=\left|{\vec{F}}_{fric}\right|\left|{\vec{r}}_{fric}\right|=\mu \;\left|{\vec{F}}_{N}\right|\;\left|{\vec{r}}_{fric}\right|=\mu \;\left|{\vec{F}}_{YZ}\right|\;\left|{\vec{r}}_{fric}\right|$$ (3)

$$\mu =\left|M_{R}\right|/\left(\left|{\vec{F}}_{YZ}\right|\;\left|{\vec{r}}_{fric}\right|\right)=\left|\left({-M}_{x}-r_{y}F_{z}+r_{z}F_{y}\right)\right|/\left(\left|{\vec{F}}_{YZ}\right|\;\left|{\vec{r}}_{fric}\right|\right)$$ (4)

### 2.5 Data evaluation

All investigated parameters, except the coefficient of friction ( μ ), were averaged intra-individually using a dynamic time warping (DTW) approach to account for temporal variations between repeated activity cycles and to obtain representative subject-specific patterns. A detailed description of the DTW methodology has been published previously (Bender and Bergmann, 2012).

To determine the average friction coefficient behavior, all primary input parameters were first averaged intra-individually using DTW, after which the corresponding mean coefficient of friction was calculated from the temporally aligned averaged data.

The evaluation focused on phase-specific and activity-dependent changes throughout the movement cycle, including peak values, local extrema, and parameter behavior during defined movement phases of the investigated activities. Particular attention was given to the coupling between friction behavior, joint loading, and knee joint kinematics, enabling a consistent phase-based comparison across subjects and activities.

## 3 Results

**FIGURE 3:**
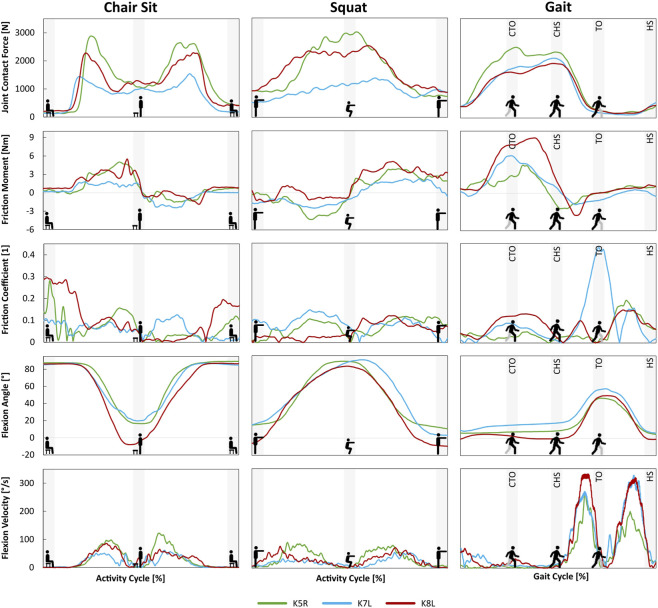
Intra-individual variability of joint contact force, friction moment, friction coefficient, knee flexion angle, and absolute knee flexion velocity during chair sit (left), squat (middle), and gait (right) for the individual participants K5R, K7L, and K8L. Specific movement events are highlighted in grey, including the start, middle, and end phases of chair sit and squat activity, as well as gait-specific events such as contralateral toe-off (CTO), contralateral heel strike (CHS), toe-off (TO), and heel strike (HS).

The intra-individual average patterns of joint contact force (Fres), friction moment (MR), friction coefficient (µ), knee flexion angle (α), and absolute knee flexion velocity (ω) for each participant are shown in Figure 3. The corresponding absolute tibiofemoral sliding velocity curves are provided in Supplementary Figure S1. The results demonstrate inter-individual variations while also revealing characteristic changes at specific movement events, including the start, middle, and end phases of the activities, as well as gait-specific events such as contralateral toe-off (CTO), contralateral heel strike (CHS), toe-off (TO), and heel strike (HS) during gait.

Overall, joint contact force and friction moment showed consistent activity-dependent patterns across subjects, whereas the coefficient of friction exhibited greater inter-individual and phase-dependent variability.

### 3.1 Chair sit activity

Based on our hypothesis that knee joint friction after total knee arthroplasty is not constant but varies with activity-dependent loading and knee joint kinematics, activities with distinct loading and kinematic profiles were included. Chair sit involved a transition from a flexed sitting position to full extension and back to sitting, reflected by a change in knee angle ranging from −8° to 90°, with distinct phases of extension and flexion.

Peak angular velocities during stand-up (extension) ranged from 50°/s ≙ 16 mm/s (K7L) to 99°/s ≙ 37 mm/s (K5R), and during sit-down (flexion) from 62°/s ≙ 24 mm/s (K7L) to 122°/s ≙ 48 mm/s (K5R). Initial and final sitting phases were characterized by low angular motion.

These resting sitting phases, characterized by low kinematic activity, were associated with low contact forces (approximately 150–400 N), whereas active movement phases (extension and flexion) showed increased loading with two consistent peaks across all subjects. The first peak occurred during rising from the chair (1440 N in K7L to 2880 N in K5R), followed by a reduction to approximately 800 (K7L) to 1000 N (K5R) during the intermediate standing phase, and a second peak during sit-down (1540 N in K7L to 2650 N in K5R) (Figure 3).

The friction moment (MR) showed positive values during extension and negative values during flexion according to the defined sign convention. Peak values ranged from 1.8 Nm (K7L) to 5.5 Nm (K8L) during extension and from −1.5 Nm (K5R) to −2.4 Nm (K7L) during flexion. In the initial and final sitting phases, friction moments remained low, with maxima up to 0.9 Nm (K8L). Overall, contact force and friction moment showed a coordinated and reproducible temporal pattern across subjects, while the coefficient of friction displayed the highest inter-individual variability within the activity cycle.

The coefficient of friction (µ) showed the highest temporal variability across the activity cycle. During the initial sitting phase, elevated values were observed in K5R and K8L (up to 0.3), while K7L remained at approximately 0.1. With the onset of extension, µ decreased in all subjects to values between approximately 0.09 (K7L) and 0.15 (K5R). During subsequent flexion, µ remained low in K5R and K8L, whereas K7L showed a moderate increase up to approximately 0.13. In the final sitting phase, µ decreased in K7L, while K5R and K8L increased again to approximately 0.12 and 0.19, respectively.

Overall, while all parameters showed comparable qualitative temporal patterns across subjects, the coefficient of friction exhibited the most pronounced inter-individual differences. This indicates that, compared to contact force and friction moment, µ is more sensitive to phase-dependent and subject-specific conditions during chair sit.

### 3.2 Squat activity

As a contrast to chair sit, a second high-flexion activity, squat, was analyzed. Similar distinct flexion and extension phases were observed, with a comparable range of motion between −9.3° (K8L) and 90.3° (K7L), although occurring in a reversed sequence. The squat movement started from a standing position, followed by a flexion phase to maximum achievable knee flexion and a subsequent extension phase returning to standing. Peak flexion velocities ranged from 33°/s ≙ 14 mm/s (K7L) to 90°/s ≙ 40 mm/s (K5R), while peak extension velocities ranged from 55°/s ≙ 18 mm/s (K7L) to 79°/s ≙ 32 mm/s (K5R), and were therefore slightly lower compared to chair sit activity.

In contrast to chair sit, elevated joint contact forces were already present during the initial and final standing phases of the squat cycle. Mean contact forces ranged from 521 N (K7L) to 937 N (K8L) in the initial standing position and from approximately 740 N (K5R) to 880 N (K8L) after returning to full extension in the final standing phase. During the pronounced flexion and extension phases, contact forces increased continuously throughout flexion, reaching peak values between 1385 N (K7L) and 3000 N (K5R).

During the flexion phase of the squat activity, the friction moment (MR) increased negatively, reaching peak values between −1.45 Nm (K8L) and −4.3 Nm (K5R). During the subsequent extension phase, MR changed to positive values with peaks ranging from 2.2 Nm (K7L) to 5 Nm (K8L). Compared to chair sit, higher friction moments were observed during flexion, while extension moments were of comparable magnitude.

During the flexion phase, the coefficient of friction (µ) was elevated in K5R (0.11) and K7L (0.15), whereas K8L remained comparatively stable at lower values, with maxima around 0.03. The µ curves during flexion showed a qualitatively similar pattern in K5R and K7L. During the subsequent extension phase, comparable peak values were observed across all participants, ranging from 0.11 (K7L) to 0.12 (K8L), and all subjects exhibited a qualitatively similar µ curve pattern. In contrast to chair sit, these patterns showed reduced inter-individual variability and no pronounced increases in µ during the initial or final movement phases. Overall, the coefficient of friction demonstrated lower inter-individual variability than during chair sit, particularly during the extension phase.

### 3.3 Gait activity

In contrast to the high-flexion activities chair sit and squat, gait represents a more dynamic daily activity with distinctly different kinematic characteristics. This is reflected in a more asymmetric flexion–extension pattern over the gait cycle, with relatively small angular changes during stance phase (from initial contact to contralateral heel strike, CHS) and more pronounced changes during the transition to swing phase (between CHS and heel strike, HS). Overall, the range of motion was considerably smaller (approximately −2° to 57°) compared to chair sit and squat.

In addition, flexion–extension velocities were substantially higher than in the two high-flexion activities, as angular changes occurred over a shorter time period. During stance phase, only small variations in knee angle were observed, with K5R and K7L maintaining a more flexed posture and flexion–extension velocities reaching up to 67°/s ≙ 47 mm/s (K8L). Shortly before toe-off (TO), a pronounced flexion phase occurred, with peak velocities increasing to approximately 265°/s ≙ 141 mm/s (K5R) to 330°/s ≙ 195 mm/s (K8L). The subsequent extension phase during swing phase, within a similar angular range, showed velocities between approximately 199°/s ≙ 109 mm/s (K5R) and 326°/s ≙ 167 mm/s (K7L). Overall, these findings underline the distinctly more dynamic kinematic behavior of gait compared to chair sit and squat.

Compared to the high-flexion activities, joint contact forces during gait were elevated primarily during the low-flexion stance phase rather than during phases of high knee flexion, as observed in chair sit and squat. Although the force magnitudes were comparably high, their temporal distribution throughout the movement cycle differed substantially. During stance, two characteristic force peaks were observed: the first at contralateral toe-off (CTO) and the second at contralateral heel strike (CHS). The first peak ranged from an average of 1614 N (K8L) to 2488 N (K5R), while the second ranged from 1918 N (K8L) to 2323 N (K5R). Between CHS and ipsilateral toe-off (TO), contact forces decreased as the knee entered a rapid flexion phase. Forces remained reduced throughout the subsequent swing phase, which was characterized by high extension velocities, before increasing again with the onset of the next stance phase at heel strike (HS).

The average friction moment (MR) increased during the stance phase and reached peak values between CTO and CHS ranging from approximately 4.8 Nm (K5R) to 8.9 Nm (K8L). After CHS, during the subsequent high-flexion phase, MR decreased to minima between approximately −1.8 Nm (K7L) and −3.5 Nm (K8L). Thereafter, the friction moment increased again and remained relatively stable throughout the swing phase, reaching values up to 1.3 Nm (K8L).

At the onset of the gait cycle, shortly after the previous heel strike (HS), the mean coefficient of friction (µ) started at relatively low values, with maxima up to 0.045 for K5R. During stance, between CTO and CHS, µ increased in K7L (up to 0.09) and K8L (up to 0.13), whereas K5R initially decreased before increasing again to approximately 0.05. Before CHS, all coefficients decreased; however, K5R showed a brief secondary increase to approximately 0.03. In contrast, K8L increased further to approximately 0.1, while K7L exhibited a pronounced rise, reaching a peak value of 0.43 at TO, after K5R and K8L had already decreased. During the subsequent swing phase, µ increased again in K5R and K8L, whereas K7L initially decreased before rising again. The highest mean coefficients during swing reached approximately 0.15 (K8L) and 0.19 (K5R).

Overall, while joint contact force, friction moment, and kinematics showed consistent temporal patterns across subjects, the coefficient of friction exhibited pronounced phase-dependent inter-individual variability, particularly between CHS and HS. A comparable behavior, with more pronounced phase-dependent inter-individual variability in µ than in the remaining parameters, was also observed during chair sit and squat activities.

Across all activities, joint contact force, friction moment, and knee kinematics showed reproducible and qualitatively similar temporal patterns between subjects, whereas the coefficient of friction demonstrated higher inter-individual and phase-dependent variability, particularly during phases of low loading and rapid kinematic transitions.

## 4 Discussion

This study provides novel insight into *in vivo* friction variability following total knee arthroplasty (TKA) under consideration of subject-specific internal loading and knee kinematics. In agreement with our hypothesis that joint friction is a dynamic, activity-dependent parameter influenced less by the load magnitude but more by the joint kinematics of the knee, the *in vivo* friction exhibited a highly dynamic behavior with characteristic variations across and throughout each analyzed activity. Maybe the lack of consistency specifically in the friction parameter itself suggests that other aspects beyond the load and movement related ones, such as lubrication-related mechanisms contribute to a relevant degree to the detected friction behavior.

During chair sit, pronounced unloading phases occurred in the initial and final parts of the movement (relaxed sitting position), providing an opportunity to evaluate lubrication recovery. Joint contact forces during chair sit were generally similar throughout flexion and extension but exhibited inter-individual differences, likely related to body weight and muscle activation. In contrast, during squat, joint contact forces remained high throughout flexion and extension. The movement started from a preloaded state, and the sustained loading throughout the activity cycle may have contributed to limited inter-articular fluid replenishment and higher friction. This distinction between the unloaded initiation of chair sit and the preloaded condition during squat may help explain the observed differences in friction behavior between the two activities.

During level walking, joint contact forces in the stance phase were comparable to previous *in vivo* TKA measurements (Kutzner et al., 2010) and to those acting on instrumented hip implants. Slightly lower forces were present during the swing phase (Damm et al., 2021), which may facilitate joint relubrication. Joint contact forces during gait were lower for K5R and K8L, but higher for K7L compared to chair sit and squat, likely reflecting differences in muscular recruitment and whole-body kinetics.

Chair sit and squat exhibited a comparable range of motion and similar flexion-extension velocities during the main movement phases. The corresponding estimated tibiofemoral sliding velocities were also comparable to previously reported *in vivo* measurements (Guan et al., 2024). However, chair sit additionally includes pronounced initial and final phases characterized by very low flexion–extension velocities. At these low velocities, synovial fluid exhibits high viscosity (Cooke et al., 1978; Schurz and Ribitsch, 1987). The squat begins from full extension, followed by maximal active flexion and a return to extension, without distinct low-velocity phases. In contrast, level walking is characterized by a smaller range of motion but much higher flexion and extension velocities, particularly during the second half of the gait cycle, reflecting a more dynamic movement pattern. Despite a different flexion–extension kinematic pattern, the peak velocity magnitudes during the second half of the gait cycle were comparable to those reported for *in vivo* hip measurements (Damm et al., 2021). The corresponding estimated peak tibiofemoral sliding velocity magnitudes were consistent with previously reported *in vivo* and *in vitro* measurements (Guan et al., 2024; Guan et al., 2017; Dumas et al., 2020; Johnson et al., 2001).

During the loaded extension and flexion phases of the chair sit movement, all participants exhibited coefficients of friction within the range expected for dry to mixed lubrication regimes when compared to *in vitro* measurements (Galetz et al., 2010). Similarly, during squat, µ values reached up to 0.15 during flexion and extension, also consistent with mixed or dry Coulomb friction observed *in vitro* (Galetz et al., 2010). While these values fall within the typical dry-to-mixed range, the detailed coefficient curves were subject-specific and exhibited inter-individual differences.

In contrast to the loaded flexion and extension phases, K5R and K8L exhibited increased µ values during the initial and final unloaded phases of chair sit, exceeding the expected range for pure Coulomb friction. This suggests contributions from fluid-film shear stresses could have occurred. It should be noted that the calculated coefficient of friction reflects not only Coulomb friction at the articulating surfaces but also viscous shear forces within the lubricant film. Flexion–extension velocities during the unloaded phase were very small. At these low velocities, synovial fluid exhibits high viscosity (Cooke et al., 1978; Schurz and Ribitsch, 1987), resulting in elevated shear stresses that could manifest as a higher measured friction coefficient. Here, subsequent increases in contact force may expel the synovial fluid from the joint, indicating that lubrication is restored only when joint loading decreases toward the end of the movement.

In contrast to that, squat activity begins in a preloaded state, likely reducing fluid-film thickness before movement initiation. The sustained loading throughout the activity cycle may further contribute to slightly higher friction moments compared to chair sit. These findings are consistent with increased friction under conditions that may correspond to predominantly boundary or mixed lubrication regimes, as synovial fluid may have been expelled from the joint gap and could not return due to the presence of pronounced loading. Similar effects have been reported *in vivo* for the hip, both during walking after short rest periods (Damm et al., 2017) and certain physical activities (Bergmann et al., 2018), supporting the generalizability of this mechanism.

During level walking, the elevated friction moment during stance was accompanied by mean peak friction coefficients of approximately 0.05–0.13, which may indicate a mixed or dry friction regime according to *in vitro* tests (Galetz et al., 2010; Henke et al., 2026). This increased friction might well have been caused by a reduced fluid film, as synovial fluid is squeezed out of the joint gap during the highly loaded stance phase, as previously suggested for the hip (Damm et al., 2013; Ga et al., 2011; Meyer and Tichy, 2003; Unsworth, 1991). In the subsequent flexion phase, the coefficient for K5R and K8L remained within a dry or mixed friction state, whereas for K7L the coefficient increased to values above 0.4, exceeding those reported *in vitro* even under lubricant-free conditions. This may indicate a transition from dry or mixed friction toward a more lubricated state associated with the formation of a thicker fluid film. According to the Stribeck curve, friction coefficients in lubricated regimes can increase with decreasing load, increasing fluid viscosity, and increasing relative sliding velocity (Stribeck and Schröter, 1903). During this phase, joint forces decreased while flexion velocities increased in all participants. *In vitro* viscosity measurements indicate a reduction in viscosity at higher velocities (Cooke et al., 1978; Schurz and Ribitsch, 1987). These conditions may favor increased fluid contribution within the articulating interface. These observations suggest that the measured coefficient of friction *in vivo* may not solely reflect dry surface interaction, but also viscous shear contributions within the lubricant layer. While such an increase could theoretically occur in all participants, this lubrication-related effect appeared less pronounced in participants K5R and K8L. These inter-individual differences may reflect variations in joint kinematic, muscle activation patterns, joint loading distribution or synovial fluid composition and surface properties. They are more likely to represent genuine subject-specific mechanical and lubrication-related behavior than methodological uncertainty, as the ultra-congruent implant geometry limits anterior-posterior translation and the smoothly varying femoral condylar radii produce only gradual changes in the friction lever arm, making it unlikely that the observed peaks in the coefficient of friction arise from the geometric approximation. The low fluoroscopic registration error further minimizes uncertainty in the estimated contact point location. During the subsequent swing phase, increasing µ values were observed in all participants under conditions of reduced joint loading and elevated angular velocities, which may indicate increasing fluid contribution within the articulating interface. Comparable observations have previously been reported *in vivo* for instrumented total hip implants (Damm et al., 2021; Damm et al., 2013).

The observed activity- and phase-dependent friction behavior may have important implications for current *in vitro* and computational approaches used to investigate TKA tribology. Existing TKA simulator protocols and *in silico* models commonly apply simplified loading conditions, standardized motion cycles, and constant friction coefficients (Galetz et al., 2010; Mazzucco and Spector, 2004; Scholes and Unsworth, 2006; Godest et al., 2002; Halloran et al., 2005; K et al., 2007; Mell et al., 2018; O’Brien et al., 2013). In contrast, the present *in vivo* measurements inherently reflect subject-specific joint kinematics and native synovial lubrication conditions. The findings therefore suggest that friction behavior may vary substantially throughout the movement cycle and could be influenced by changing loading and motion conditions. In particular, the pronounced phase-dependent variability of µ indicates that friction behavior *in vivo* may not be fully represented by static or constant friction assumptions alone. Incorporating more dynamic and subject-specific friction characteristics into preclinical testing and computational simulations may improve physiological relevance and predictive accuracy.

### 4.1 Limitation

Although the presented approach is unique, it is based on a very small cohort of only three subjects implanted with an ultra-congruent TKA system, with an unequal distribution of men and women and only a few repetitions per activity. Consequently, the statistical power is limited, and the findings should be interpreted with caution. Furthermore, the investigated TKA system features an ultra-congruent bearing geometry, which is now largely considered outdated compared with the more forgiving implant geometries currently used in clinical practice nowadays. As a result, the findings may not be generalizable to other implant designs. Future studies including a broader range of implant geometries are therefore needed to determine the wider applicability of the observed friction behavior. Nevertheless, this dataset remains unique, as it is the only one currently available that combines synchronized *in vivo* joint loading with fluoroscopic kinematics, providing valuable information for future TKA design evaluation and development. In addition, the friction analysis considers only motion in the sagittal plane, and the contact point location is approximated. Consequently, the proposed approach cannot capture potential friction effects arising from compartment-specific loading and contact kinematics, including compartment-specific sliding velocities, axial rotation, or varus–valgus motion. Although these contributions are expected to be small for the investigated ultra-congruent implant, owing to its symmetrical polyethylene insert and highly constrained kinematics, they may become more relevant for asymmetrical or less constrained implant designs.

## 5 Conclusion

This study provides the first *in vivo* characterization of friction after total knee arthroplasty (TKA) while accounting for subject-specific joint kinematics. Friction was shown to be highly dynamic and activity-dependent, likely reflecting the combined influence of loading conditions, joint motion, and possible lubrication-related effects. Unloading phases, such as during chair sit and gait swing, may facilitate fluid replenishment within the articulating interface, whereas sustained loading, as observed during squatting, was associated with higher friction behavior consistent with mixed or boundary lubrication conditions.

From a clinical perspective, elevated friction forces may increase mechanical stress on the implant inlay surface, potentially contributing to wear, micromotion at the implant–bone interface, and ultimately aseptic loosening. Incorporating *in vivo* friction characteristics into preclinical testing, implant design, and *in silico* models may improve physiological relevance and predictive accuracy, particularly by considering friction as a non-constant and potentially velocity-dependent parameter.

These findings demonstrate that *in vivo* knee joint friction after TKA is dynamic and depends on both activity type and motion phase. The observed friction dynamics illustrates that it is only partially linked to contact load magnitude, whereas motion velocity and lubrication-related mechanism appears to substantially influence the frictional response. These findings may support the refinement of experimental and computational models used in TKA tribology and implant evaluation.

## Data Availability

The primary data used in this study is publicly available and can be requested from https://cams-knee.orthoload.com. Requests to access the datasets should be directed to Philipp Damm, philipp.damm@charite.de.
