# Supplementary material for: In vivo knee joint friction after total knee arthroplasty is highly dynamic and phase-dependent
Source: Front Bioeng Biotechnol. 2026 Aug 4;14:1885711. doi: 10.3389/fbioe.2026.1885711 (PMC13481440; doi:10.3389/fbioe.2026.1885711)
Supplement: Supplementary file 1 [file Supplementaryfile1.docx]

Supplementary Material

**Supplementary Figure 1.** Intra-individual variability of absolute tibiofemoral sliding velocity during chair sit (left), squat (middle), and gait (right) for the individual participants K5R (green), K7L (blue), and K8L (red).
